# Diagnosing Frailty in Primary Care Practice

**DOI:** 10.7759/cureus.23329

**Published:** 2022-03-19

**Authors:** Manoj R Somagutta, Utkarsha Uday, Narayana R Bathula, Siva Pendyala, Ashwini Mahadevaiah, Molly S Jain, Greta Mahmutaj, Mohamed Gad, Jennifer Jean Baptiste

**Affiliations:** 1 Department of Medicine and Research, Avalon University School of Medicine, Willemstad, CUW; 2 Department of Research, California Institute of Behavioral Neurosciences & Psychology, Fairfield, USA; 3 Department of Medicine, West Bengal University of Health Sciences, Kolkata, IND; 4 Department of Medicine, JSS Academy of Higher Education and Research, Mysore, IND; 5 Department of Medicine, Saint James School of Medicine, Windsor, CAN; 6 Department of Medicine, University of Medicine Tirana, Tirana, ALB; 7 Department of Family and Community Medicine, St. George's University School of Medicine, St. George's, GRD; 8 Department of Medicine, Saint James School of Medicine, Park Ridge, USA

**Keywords:** family practice, screening tools, geriatric, frailty, fragility, elderly, disability, aging

## Abstract

Frailty is a complex age-related clinical condition with increased vulnerability to negative health outcomes that manifest as a multidimensional syndrome and hence, a challenge to identify at-risk populations. We aim to summarize the implementation of strategies to diagnose fragility in family practice using current evidence. We searched the PubMed and Google Scholar databases for relevant articles, using the Medical Subject Headings (MeSH) terms "Frailty," "Frailty Scales," and "Primary Health Care." All original research articles on the elderly population (65 years of age or older) published in English and the last five years were included. Frailty diagnosis has resulted in positive outcomes in the overall literature. Recent hospital admission may indicate a health problem that can end up in a negative outcome and has been often described as associated with frailty. It was also shown to affect the intensive care units' mortality, in-hospital mortality, and long-term mortality. However, multiple screening instruments have been developed and validated to improve feasibility in clinical practice. The frequent lack of agreement between frailty instruments has slowed the broad implementation of these tools. The impacts of frailty warrant an upstream, proactive, holistic, interprofessional primary care approach to its identification, assessment, and management. It is a preventable disorder; identifying elderly patients at risk in primary care can help shape appropriate care processes tailored to their needs. This literature review aims to demonstrate the importance and strategies in identifying frailty in primary care settings and assess its impact on several outcomes.

## Introduction and background

The aging process is a natural biological phenomenon involving physical, mental, and social changes. It is influenced by an individual's social, political, and economic factors [1]. Life expectancy has increased in developed countries, reaching a mean age of 83 years, thereby increasing the proportion of elderly with any disability [2].

According to physicians, older adults exhibit a specific vulnerability. This state of vulnerability, stemming from impairments in multiple organ systems, leads to increased susceptibility to poor health outcomes [3]. It is a multifactorial and complex clinical syndrome where a patient has incremental vulnerability to disability. Prior to a disability, an intermediate state known as frailty is characterized by diminished capacity to respond to stressors due to a reduced functional reserve [2]. Frailty prevalence rises with advancing age, from 16% in people older than 65 to as high as 52% in those older than 85 [4]. Frailty syndrome encompasses physical, cognitive, psychological, and social factors. It is often seen as a bridge between healthy aging and disability. In a systematic review, the prevalence of falls in the frail elderly was reported to be between 6.7% and 44% [5]. Over half of the frail adults are reported to have fallen in the previous year [6]. Frailty-induced falls are associated with a greater risk of fractures, disability, immobility, hospitalizations, institutionalization, caregiver burden, decreased life quality, and even death [4,7]. 

Although physical activity, nutrition, memory training, and the comprehensive geriatric assessment have been explored as single or multicomponent interventions, none of these are specific to frailty [3]. The lack of evidence for clinical decision-making and costs related to screening for frailty in primary care does not improve clinical outcomes. This literature review aims to demonstrate the importance and strategies in identifying frailty in primary care settings and assess its impact on several outcomes associated with mortality. 

## Review

Methods

We searched the PubMed and Google Scholar databases for relevant articles, using the Medical Subject Headings (MeSH) terms "Frailty," "Frailty Scales," and "Primary Health Care." All original research articles on the elderly population (65 years of age or older) published in English and the last five years were analyzed. Articles published in languages other than English were not considered. 

Results

Fragility diagnosis has resulted in positive outcomes in the overall literature. Recent studies have associated frailty with increased intensive care unit (ICU) mortality (RR 1.51, 95% CI 1.31-1.75), in-hospital mortality (RR 1.71, 95% CI 1.43-2.05), and long-term mortality (RR 1.53, 95% CI 1.40-1.68), independent of the severity of illness and other prognostic indicators [6-8]. Recent hospital admission may indicate a health problem that can result in a negative outcome and has been often described as associated with frailty. A high association between the two was found in an article by Diez-Ruiz et al. (p=0.002) [9]. It has been noticed that frail elderly patients are more likely to die or be readmitted within 30 days to six months after the surgical procedure [10]. Comprehensive geriatric assessment (CGA) resulted in fewer ER admissions (2.6% to 19.7%) [8,10]. The clinical frailty scale (CFS), physical performance tools, and patient questionnaires are relevant and straightforward tools appropriate in clinical practice (Table 1).

**Table 1:** Common frailty screening tools in primary care settings ADLs = activities of daily living; IADLs = instrumental activities of daily living; SHARE = Survey of Health, Ageing and Retirement in Europe

Common frailty screening tools in primary care settings			
Tool	Type	Components	Concepts
FRAIL scale	Five-item questionnaire for frailty identification.	Fatigue, resistance, ambulation, illnesses, loss of weight	Disability, mortality, ADLs, IADLs
Clinical Frailty Scale (CFS)	The nine-point scale with a descriptor of a frailty stage.	Very fit; well; managing well; vulnerable' mildly, moderately, severely or very severely frail; terminally ill	Fatigue, fitness, comorbidities, disability
Vulnerable Elders Survey (VES-13)	13-item survey to assess elderly vulnerability.	Age, self-rated health limitations in physical function	Vulnerability, physical activity
SHARE Frailty Instrument (SHARE-FI)	Mixed questionnaire and performance-based instrument.	Exhaustion, weight loss, weakness, slowness, low activity	ADLs, physical activity, physical strength, mortality
Folstein Mini-Mental State (MMS)	30-point questionnaire to measure cognitive impairment.	Orientation, immediate recall, attention and calculation, recall, language	Cognition, mental function, memory
Lubben Social Network Scale	12-item scale to assess social isolation in older adults	Family, friendships	Social activities, support

Besides validity and reliability in a busy primary care setting, other factors such as ease of use (based on time, training, and equipment) may affect a practitioner's choice of frailty measures [7]. Some of the standard assessment tools used in clinical practice are judgment-based tools (e.g., the Clinical Frailty Scale), physical performance tools (e.g., gait speed), patient questionnaires (e.g., Program of Research to Integrate the Services for the Maintenance of Autonomy [PRISMA-7]), the electronic frailty index and multidimensional measures (e.g., Edmonton Frail Scale) [7]. These indices assess deficits in areas such as strength, balance, nutrition, resistance, mobility, physical activity, and cognitive ability. Still, none of them have been taken up in primary care settings [7]. Few available multidimensional tools can be used for both cases finding and component definition (i.e., exploring the components of frailty such as cognitive ability, multimorbidity, functional status and mobility, polypharmacy, social supports, continence, and mood) [7]. However, no specific instruments or biomarkers are adequately proven to be superior to others [4,7].

Discussion

Aging or senility is a progressive phenomenon in which different biopsychosocial changes occur from birth till death. Frailty has been confused with the phrases aging, disability, and comorbidity in the past. However, there are apparent differences between them [11]. Some of the differences are as outlined. Advanced aging does not necessarily make the elderly prone to adverse outcomes, which is typical of frailty [12]. Frailty is associated with lower socioeconomic status and could be programmed in early life depending on the patient's circumstances [13]. Frailty has been thought to be decreased physiological reserve and limited capacity to maintain normal bodily functions and homeostasis. However, frail older people can develop adverse health outcomes due to internal and external stress factors [14].

Moreover, frailty refers to the risk involved in losing functions and instability. In contrast, disability indicates loss of function involving difficulty in completing tasks related to activities of daily living (ADLs) such as bathing, dressing, eating, toileting, and continence. This also involves a loss in the functioning of instrumental activities of daily living (IADLs) such as meal preparation, housekeeping, laundry, transportation, telephone use, etc. [15]. Furthermore, comorbidity is a term used to describe a patient with medically diagnosed two or more conditions distinguishable from frailty [11].

The association of such changes with comorbidities facilitates the appearance of geriatric syndromes [1]. Within the last 20 years, geriatric medicine experts have made significant efforts to identify older people requiring specialized attention that may delay or avoid the appearance of disability and its intermediate state, frailty [2]. The Frailty Syndrome in the geriatric population is considered multidimensional. The dysfunction of one of the dimensions harms the other. This generates a cascade, which potentiates the appearance of adverse effects, such as falls, hospitalizations, geriatric disabilities, chronic diseases, and metabolic disorders [1].

Obesity, insulin resistance, arterial hypertension, decreased physical performance are shared among the geriatric population. The various medical conditions that a patient has are informative of the overall health state, so it is routinely collected as part of geriatric assessments. For instance, sarcopenia leads to changes in muscle mass and an increase in body fat percentage due to senile slowing of metabolism [1,2]. The falls of the elderly affect the family, social and economic dynamics because they increase the number of hospitalizations due to fractures, can generate incapacities, and lead to premature death. Few identifiable risk factors for falls in the elderly are impaired vision, pain, gait alteration, lack of physical conditioning, polypharmacy, comorbidities, impaired balance, musculoskeletal impairment, sarcopenia, pre-obesity, obesity, decreased cognitive function, and sedentary lifestyle [1-4]. Also, with aging, the information processing capacity decreases. The decline of cognition affects autonomy, decision-making, independence, and one's possibility of performing activities of daily living without the help of others that affects the family environment in which this older adult is residing, and all these elements are risk factors for abandonment [1].

This scoping review provides essential information on the impact of frailty syndrome on self-health and clinical outcomes in the elderly. It emphasizes clinical methods and tools to render the diagnosis in the primary care settings and focuses on identifying patients at risk, approaches to treat the disease, and effective sub-specialty evaluations.

**Figure 1:**
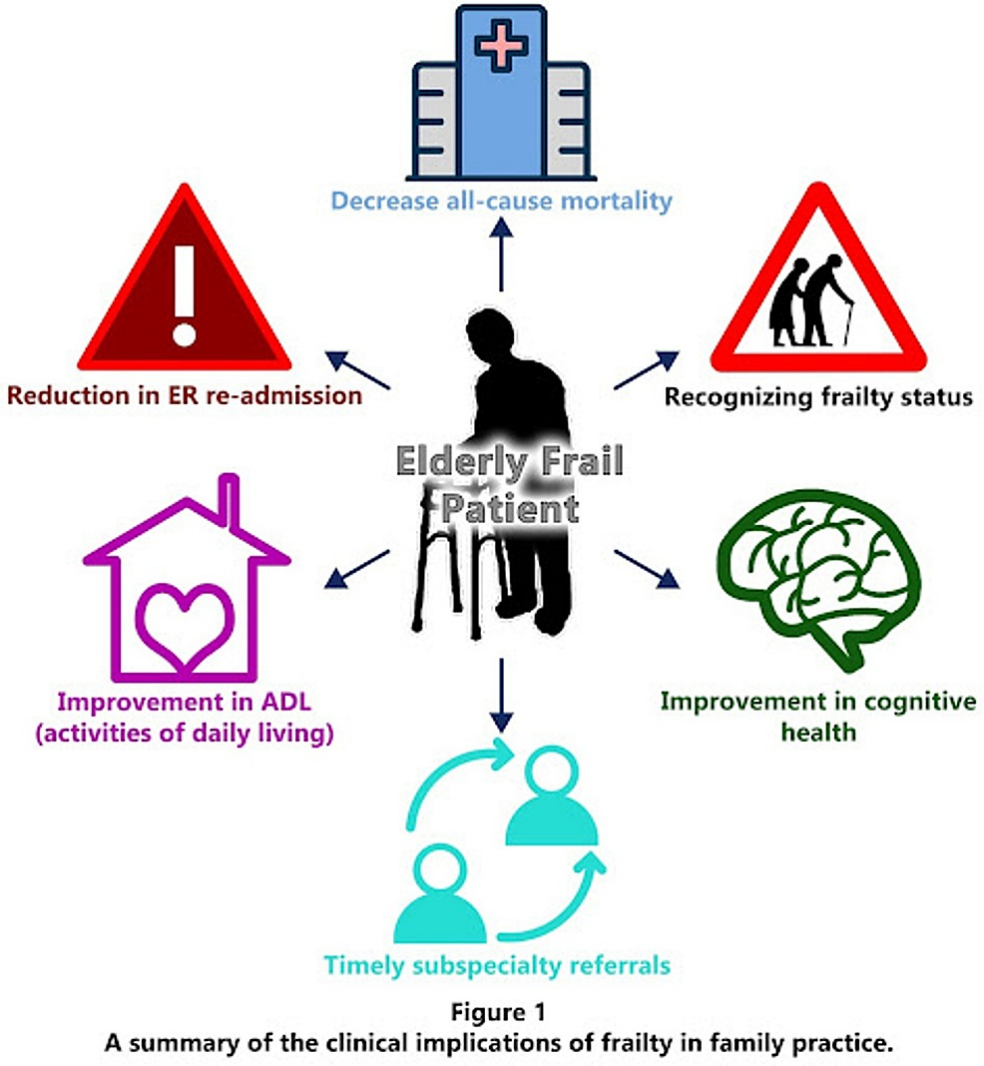
Clinical implications of frailty in primary care Original figure made by the authors

Frailty is the leading risk factor for the appearance of disability, which is unlikely to recover. However, we can potentially reverse frailty with lifestyle modifications [2]. Possible strategies to prevent frailty include lifestyle/behavioral factors such as exercise, proper nutrition, and cognitive health maintenance [4]. Awareness camps and community-based programs screening for the vulnerable will go a long way. Geriatric patients may be referred to a geriatrician for additional concerns (Figure 1).

At present, the health care system is more designed to address disease-specific and organ-specific needs for the patients but not well equipped to meet the needs of the complexity of frail patients [16]. Hence, older patients are susceptible to obtaining suboptimal patient care with fragmented treatment and healthcare services [17]. The primary direction on frailty management in elderly patients in primary care requires an intensive, multidisciplinary approach involving geriatricians, allied health professionals (including physiotherapists, dieticians, exercise physiologists, social workers, and occupational therapists), caregivers, and the patient themselves [18]. Using this principle, the patient-centered medical home model (PCMH) in the US can allow safe, patient-centered, accessible, and high-quality care of elderly patients with frailty [19]. A study in Singapore demonstrated the impact of a patient-centered medical home model on quality of life and patient activation for older adults with frailty and complex needs. After analyzing 165 elderly patients, they noticed that the PCMH showed improved needs satisfaction, better access to care, quality of life (QoL), and patient activation [20]. This model provides a better structure for primary care providers to implement screening and management strategies [21]. The Chronic Care Model (CCM) may also drive quality improvement measures for primary care delivery. When assessed objectively, the implementation of interventions in the CCM dimension improved the quality of primary care as perceived by general practitioners [22]. It further enhances primary care providers to effectively work in teams and referrals as needed to appropriate specialty for ensuring optimal elderly care for those diagnosed with frailty. However, structural financing and manpower and the availability of a facilitating information and communication technology system are significant challenges to implementing and embedding this approach.

Despite being a good indicator of poor outcomes, frailty has limited evidence-based screening tools. Many studies report a variety of frailty scales, but their reliability and validity have seldom been studied [23]. Bouillon and colleagues highlight that only a few studies have evaluated frailty scales for reliability and validity or following specific standards. Acceptable reliability coefficient and predictive validity have been confirmed for the Clinical Frailty Scale and the Edmonton Frail Scale. The frailty index and the Fried scale have been tested for validity but not reliability [23]. However, various anomalies (either technical, professional,l or plagiarism) may occur with many assessment scales [23]. In a population-based cross-sectional study of 214 elderly individuals, the validity of five screening methods, e.g., Clinical Frailty Scale, simple FRAIL questionnaire, PRISMA-7 questionnaire, Timed Up and Go Test (TUG), and Gérontopôle Frailty Screening Tool (GFST) was assessed [24]. The study reported the prevalence of frailty in the community was 11.7% using the Fried phenotype (CHS criteria), and their findings indicate that the simple FRAIL questionnaire and GFST had the highest sensitivity compared with CHS criteria a reference standard. However, the CFS had the highest specificity making it appropriate for confirming the diagnosis because it has high specificity but low sensitivity [24]. None of the assessment scales examined are used as the gold standard in the primary care setting. Various tools are being used in clinical practice to assess frailty in elderly patients, emphasizing the need for standardization and guidelines. This requires evaluating the current examination tools for validity and reliability and appropriately enhancing them [25]. Also, the assessment tools should aim to identify a precise level of risk, beneficial for treatment [26]. 

Evidence-based guidance to support screening and management of frailty in primary care is sorely needed. Current priorities are to establish consensus on a clinical tool that provides added clinical value and assess whether interventions improve patient-centered outcomes [3]. Further research should explore the implications of frailty in younger age groups. Interventions need to be designed to identify, prevent, and manage frailty and pre-frailty across the age spectrum, particularly those with multimorbidity. There is also a pressing need for public engagement on this subject and research to explore understanding and beliefs about frailty and frailty prevention. Besides, patient- and caregiver-oriented measures, such as quality of life and function, should be incorporated as outcome measures in studies of frailty.

## Conclusions

Frailty is a clinically distinct syndrome from disability. Using specific tools and recognizing elderly patients at risk in primary care can help shape appropriate care processes tailored to their needs, besides prompt discussions with patients about their goals, objectives, preferences, and priorities for care. Lack of evidence-based guidelines and consensus on a clinical tool may be challenging. These limitations should not prohibit primary caregivers from applying clinical approaches to identify and provide patient-centered care. Proactive care delivery and multidisciplinary collaboration are paramount. The patient-centered medical home model and chronic care model can provide better access to primary care, improved quality of life, and patient satisfaction. However, there is a strong need for future research on the added clinical value, evidence-based guidelines on assessment tools, treating, and reversing risk in some patients.
